# Efficacy and Safety of Oxybuprocaine Hydrochloride Gel in Alleviating Pain during Male Urethral Catheterization: A Single-Center Randomized Controlled Study

**DOI:** 10.1155/2022/5734387

**Published:** 2022-09-14

**Authors:** Zhenkun Dong, Xutong Qu, Lu Zhang, Xueting Chen, Yuhang Dong, Hui Chen, Yan Cui

**Affiliations:** ^1^Department of Urology, Harbin Medical University Cancer Hospital, Harbin, China; ^2^Department of Anesthesiology, Harbin Medical University Cancer Hospital, Harbin, China

## Abstract

**Background:**

The purpose of this study was to determine whether oxybuprocaine hydrochloride gel could alleviate pain during male catheterization.

**Methods:**

Between September 2021 and March 2022, a randomized controlled trial was conducted at the Urology Department of Harbin Medical University Cancer Hospital (China). A total of 192 adult male patients requiring catheterization were enrolled and randomly assigned to one of two groups: 96 in the test group and 96 in the control group. The test group included patients who received oxybuprocaine hydrochloride gel as urethral lubricant, while patients in the control group received liquid paraffin. The preoperative and postoperative pain scores were compared using nonparametric tests.

**Results:**

At the baseline, there was no significant difference between the two groups. There was no significant difference in preoperative pain scores between the test group (mean ± SD = 20.04 ± 2.68 mm) and the control group (mean ± SD = 20.21 ± 3.23 mm) (*p*=0.694). Postoperative pain scores increased significantly in the test (mean ± SD = 31.98 ± 2.57 mm, *p* < 0.001) and control groups (mean ± SD = 38.96 ± 2.02 mm, *p* < 0.001) groups. Postoperative pain scores were significantly lower in the test group (mean ± SD = 31.98 ± 2.57 mm) than those in the control group (mean ± SD = 38.96 ± 2.02 mm (*p* < 0.001).

**Conclusions:**

The use of oxybuprocaine hydrochloride gel significantly reduced pain during male urethral catheterization. The study provides evidence for clinicians to use oxybuprocaine hydrochloride gel during male catheterization.

## 1. Introduction

Catheterization allows urine to drain from the bladder. Catheterization involves inserting a catheter made of latex, polyurethane, or silicone into the bladder via the urethra to drain urine [1]. Catheterization is used for various reasons, including the relief of urinary retention, diagnosis, and treatment of bladder and urethra diseases [2, 3].

The male urethra is a fibromuscular tube that drains urine from the bladder. It has a longer, more complicated curse than the female urethra, making it more susceptible to injury during catheterization. Urethral stricture, urinary incontinence, erectile dysfunction, and infertility have been reported as iatrogenic urethral catheterization injuries [4]. Despite efforts to educate and train healthcare professionals on urethral catheterization insertion techniques, iatrogenic urethral injuries continue to occur. New strategies aimed at reducing procedural pain during urethral catheterization involve the squeeze technique during insertion and local anesthetics [5, 6]. Other variables, such as the catheter size and patient age, can have an impact on the pain experience during catheterization [7, 8].

Oxybuprocaine hydrochloride is a highly permeable and rapidly absorbed anesthetic. It binds to sodium channels and reversibly stabilizes the neuronal membrane that decreases its permeability to sodium ions. When administered as gel, oxybuprocaine hydrochloride provides adequate anesthesia for diagnostic purposes and small operations. It is suitable for catheter insertion providing analgesia. Prior studies focused on the application of anesthetic gels during cystoscopy procedures [9, 10]. Only few studies used anesthetic gels during urethral catheterization. The main objective of this study was to determine the efficacy and safety of oxybuprocaine hydrochloride gel for male urethral catheterization.

## 2. Materials and Methods

This is a randomized controlled study conducted at the Urology Department of Harbin Medical University Cancer Hospital (China) between September 2021 and March 2022. The study protocol was approved by the local ethics committee. The trial was strictly designed in accordance with the CONSORT statement [11]. Patients or their legal guardians signed the informed consent in accordance with the institutional law.

We enrolled male patients over the age of 50 who required indwelling or intermittent catheterization. We collected information about patients' history of hypertension, diabetes, indwelling or intermittent catheterization, and urinary tract infection. The exclusion criteria were as follows: altered mental state, impaired vision, inability to report visual analogue scale (VAS) scores, refusal to use treatments, or suspected allergy to oxybuprocaine hydrochloride gel. A study nurse was responsible for enrolling patients after explaining the study's objectives to them. Patients who agreed to participate were randomly assigned to receive either oxybuprocaine hydrochloride gel or liquid paraffin by selecting an envelope from a box containing either the study gel or liquid paraffin. The practice nurse opened the envelope and dispensed 10 ml of the study gel or liquid paraffin onto a sterile catheter tray. Before catheterization, the study nurse scored the patients using the VAS scale (0–100 mm). The study nurse injected 5 ml of either oxybuprocaine hydrochloride gel or liquid paraffin into the urethra and applied the remaining 5 ml onto the catheter surface. Approximately 5 minutes after catheterization, subjects were scored again using the VAS scale. VAS scores of 33 mm or less were categorized as mild pain, VAS scores between 34 and 67 mm were categorized as moderate pain, and VAS scores between 68 and 100 mm were categorized as severe pain. All the procedures were performed by a single study nurse.

SPSS Statistics 20.0 was used for statistical analysis. Data were compared between groups by the independent sample *t* test and one-way ANOVA. Homogeneity of variance was tested by Levene's test. The paired sample *t* test was used for intragroup comparison. Categorical data were compared by Fisher's exact probability method. The level of significance was 0.05 (bilateral).

## 3. Results

We enrolled a total of 192 adult male patients requiring catheterization. They were randomly assigned to one of two groups: 96 patients were treated with oxybuprocaine hydrochloride gel, and 96 patients were treated with liquid paraffin. The mean age (± standard deviation) of patients in the oxybuprocaine hydrochloride gel group was 61.57 ± 4.95 years, while the mean age (± standard deviation) of patients in the control group was 61.45 ± 5.51 years (*p*=0.869). Regarding baseline characteristics, there were no significant differences between the two groups (Table 1).

**Figure 1:**
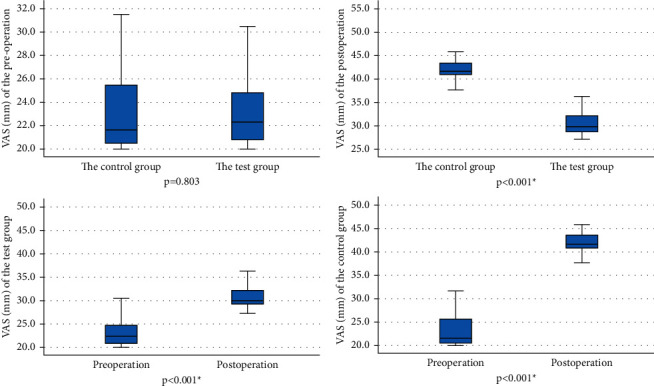
Median VAS of preoperative and postoperative scores in the test and control groups (*n* = 96 for each group).

The preoperative pain scores were not significantly different between the test (mean ± SD = 20.04 ± 2.68 mm) and control (mean ± SD = 20.21 ± 3.23 mm) groups (*p*=0.694). Postoperative pain scores increased significantly in the test (mean ± SD = 31.98 ± 2.57 mm, *p* < 0.001) and control (mean ± SD = 38.96 ± 2.02 mm, *p* < 0.001) groups. Postoperative pain scores in the test group were significantly lower than those in the control group (*p* < 0.001) (Figure 1). The administration of oxybuprocaine hydrochloride gel statistically and clinically reduced the patient's pain scores (Table 2).

There was no significance difference in catheter size distribution between the two groups (*p* > 0.05) (Table 3). No statistically significant difference was reported regarding pain scores between the two groups before and after surgery with different catheter sizes (*p* > 0.05) (Table 4).

All 96 patients of the test group were included in the safety analysis. During the study, the most common reported adverse events were itching (8.3%), erythema (7.3%), dermatitis (6.3%), and elevated blood pressure (4.2%). The adverse events disappeared in a few minutes, and no other interventions were used. No serious adverse events were observed (Table 5).

## 4. Discussion

Catheterization is widely used in hospitals, particularly, during surgical procedures when the majority of patients are awake and require preoperative indwelling catheterization [12]. However, most of them report discomfort and pain [13]. In our study, the pain scores of patients increased significantly in both groups, indicating that all patients experienced pain during catheterization. It is critical to minimize the discomfort and pain experienced during catheterization.

Regional anesthetic gels are rarely used in conventional catheterization procedures [14]. Lidocaine gel alleviated the discomfort and pain associated with catheterization, but its dosage and administration route remain disputed [15, 16]. In our study, we locally administered oxybuprocaine hydrochloride gel before catheterization. Oxybuprocaine hydrochloride was administered as homogeneous viscous gel absorbed by mucosal surfaces. Compared to the control group, the test group showed reduced postoperative pain scores, indicating that the use of oxybuprocaine hydrochloride gel alleviated pain during catheterization. Of interest, no relevant adverse events occurred in the study. The most common adverse events recovered within a few minutes. The adverse events related to lidocaine gel administration include ocular discomfort, elevated blood pressure, accelerated heart rate, paresthesia, cardiac arrest, and shock [17, 18]. Due to its safety profile, lidocaine gel is not commonly used in catheterization. Oxybuprocaine hydrochloride gel is safer than lidocaine gel.

The catheter type and catheter size may aggravate the pain sensation during catheterization [19, 20]. Larger catheters may result in more pain, discomfort, urethral irritation, and trauma [21]. However, we used four catheter sizes that did not influence the study results. A similar study found similar pain scores between different catheter sizes [22]. The role of the catheter type and catheter size in pain sensations requires further studies to validate.

Despite the study being a randomized controlled trial, we faced a number of limitations that might influence the findings. The VAS scores could not accurately reflect patients' perception of pain during the catheterization process. The majority of participants experienced pain in specific areas of the urethra; thus, mapping pain to specific areas of the urethra may increase the validity of our study. In addition, we did not check whether patients have experienced pain during previous catheterizations. They might be more tense and nervous during our investigation, affecting the individual pain scores.

## 5. Conclusions

Conventional procedures do not include regional anesthesia prior to catheterization. In this study, the application of oxybuprocaine hydrochloride gel was safe and effective in alleviating pain during male urethral catheterization.

## Figures and Tables

**Table 1:** Characteristics of the study participants.

	Test group	Control group	*p* value
Patients	96	96	1.000
Age (years)	61.57 ± 4.95	61.45 ± 5.51	0.869
Indications for catheterization			0.608
Indwelled before surgery (%)	66 (68.8)	64 (66.7)	
Urinary retention (%)	10 (10.4)	8 (8.3)	
Bladder infusion therapy (%)	20 (20.8)	24 (25.0)	
Hypertension			0.801
Yes (%)	8 (8.3)	9 (9.4)	
No (%)	88 (91.7)	87 (90.6)	
Diabetes			0.639
Yes (%)	9 (9.4)	11 (11.5)	
No (%)	87 (90.6)	85 (88.5)	
Smoking			0.810
Yes (%)	9 (9.4)	10 (10.4)	
No (%)	87 (90.6)	86 (89.6)	
History of indwelling or intermittent catheterization			0.600
Yes (%)	19 (19.8)	22 (22.9)	
No (%)	77 (80.2)	74 (77.1)	
Urinary tract infection			0.536
Yes (%)	15 (15.6)	12 (12.5)	
No (%)	81 (84.4)	84 (87.5)	
Catheterization method			0.397
Intermittent (%)	20 (20.8)	25 (26.0)	
Indwelling (%)	76 (79.2)	71 (74.0)	

^*∗*^Significant at *p* < 0.05. 192 participants were included in this study (96 patients in the test group and 96 patients in the control group). *p* < 0.05 was considered statistically significant.

**Table 2:** Comparison of pain scores between the two groups in preoperation and postoperation.

Groups	Patients	VAS score (mm)		*t*-value	*p* value
		Preoperation	Postoperation		
Test group	96	20.04 ± 2.68	31.98 ± 2.57	74.834	0.000
Control group	96	20.21 ± 3.23	38.96 ± 2.02	51.476	0.000
*t*-value	—	-0.394	-20.953		
*p* value	—	0.694	0.000		

^*∗*^Significant at *p* < 0.05. There was no statistical difference in pain scores between the two groups in preoperation and postoperation (*p* > 0.05). The pain scores of patients in the test and control groups were higher in postoperation than those in preoperation (*p* < 0.05). Postoperative pain scores were significantly lower in the test group than those in the control group (*p* < 0.05) (*n* = 96 for each group).

**Table 3:** Comparison of catheter size distribution between the two groups.

	Patients	Catheter size			
		8F	10 F	16F	18F
Test group	96	2 (50.00)	18 (43.90)	5 (62.50)	71 (51.08)
Control group	96	2 (50.00)	23 (56.10)	3 (37.50)	68 (48.92)
*p* value	—	0.781			

There was no statistical difference in catheter size distribution between the two groups (*p* > 0.05).

**Table 4:** Comparison of pain scores in patients with different sizes of catheters.

Catheter size	Patients	Pain scores	
		Preoperation	Postoperation
8F	4	21.65 ± 2.65	35.63 ± 5.44
10F	41	19.87 ± 2.72	35.70 ± 4.59
16F	8	19.04 ± 2.06	33.73 ± 3.98
18F	139	20.26 ± 3.01	35.50 ± 4.06
*F*-value	—	0.932	0.503
*p*-value	—	0.426	0.681

There was no statistical difference in pain scores of patients with different sizes of catheters in preoperation and postoperation (*p* > 0.05).

**Table 5:** Adverse events (*n* = 96 in the test group).

Adverse events	*N* (%)				
	Grade 1	Grade 2	Grade 3	Grade 4	All grades
Regional itching	5 (5.2%)	3 (3.1%)	0 (0%)	0 (0%)	8 (8.3%)
Erythema	4 (4.2%)	3 (3.1%)	0 (0%)	0 (0%)	7 (7.3%)
Dermatitis	4 (4.2%)	2 (2.1%)	0 (0%)	0 (0%)	6 (6.3%)
Elevated blood pressure	3 (3.1%)	1 (1.0%)	0 (0%)	0 (0%)	4 (4.2%)
Dizziness	2 (2.1%)	3 (3.1%)	0 (0%)	0 (0%)	5 (5.2%)
Headache	0 (0%)	1 (1.0%)	0 (0%)	0 (0%)	1 (1.0%)

Adverse events were reported in 96 patients in the test group after using oxybuprocaine hydrochloride gel.

## Data Availability

The data are included in the article and can be obtained from the corresponding authors upon request.
